# Key challenges for delivering clinical impact with artificial intelligence

**DOI:** 10.1186/s12916-019-1426-2

**Published:** 2019-10-29

**Authors:** Christopher J. Kelly, Alan Karthikesalingam, Mustafa Suleyman, Greg Corrado, Dominic King

**Affiliations:** 1 Google Health, London, UK; 2 DeepMind, London, UK; 3 Google Health, California, USA

**Keywords:** Artificial intelligence, Machine learning, Algorithms, Translation, Evaluation, Regulation

## Abstract

**Background:**

Artificial intelligence (AI) research in healthcare is accelerating rapidly, with potential applications being demonstrated across various domains of medicine. However, there are currently limited examples of such techniques being successfully deployed into clinical practice. This article explores the main challenges and limitations of AI in healthcare, and considers the steps required to translate these potentially transformative technologies from research to clinical practice.

**Main body:**

Key challenges for the translation of AI systems in healthcare include those intrinsic to the science of machine learning, logistical difficulties in implementation, and consideration of the barriers to adoption as well as of the necessary sociocultural or pathway changes. Robust peer-reviewed clinical evaluation as part of randomised controlled trials should be viewed as the gold standard for evidence generation, but conducting these in practice may not always be appropriate or feasible. Performance metrics should aim to capture real clinical applicability and be understandable to intended users. Regulation that balances the pace of innovation with the potential for harm, alongside thoughtful post-market surveillance, is required to ensure that patients are not exposed to dangerous interventions nor deprived of access to beneficial innovations. Mechanisms to enable direct comparisons of AI systems must be developed, including the use of independent, local and representative test sets. Developers of AI algorithms must be vigilant to potential dangers, including dataset shift, accidental fitting of confounders, unintended discriminatory bias, the challenges of generalisation to new populations, and the unintended negative consequences of new algorithms on health outcomes.

**Conclusion:**

The safe and timely translation of AI research into clinically validated and appropriately regulated systems that can benefit everyone is challenging. Robust clinical evaluation, using metrics that are intuitive to clinicians and ideally go beyond measures of technical accuracy to include quality of care and patient outcomes, is essential. Further work is required (1) to identify themes of algorithmic bias and unfairness while developing mitigations to address these, (2) to reduce brittleness and improve generalisability, and (3) to develop methods for improved interpretability of machine learning predictions. If these goals can be achieved, the benefits for patients are likely to be transformational.

## Background

The exciting promise of artificial intelligence (AI) in healthcare has been widely reported, with potential applications across many different domains of medicine [1, 2]. This promise has been welcomed as healthcare systems globally struggle to deliver the ‘quadruple aim’, namely improving experience of care, improving the health of populations, reducing per capita costs of healthcare [3], and improving the work life of healthcare providers [4].

Nevertheless, the potential of AI in healthcare has not been realised to date, with limited existing reports of the clinical and cost benefits that have arisen from real-world use of AI algorithms in clinical practice. This article explores the main challenges and limitations of AI in healthcare, and considers the steps required to translate these potentially transformative technologies from research to clinical practice.

### The potential of artificial intelligence in healthcare

A rapidly accelerating number of academic research studies have demonstrated the various applications of AI in healthcare, including algorithms for interpreting chest radiographs [5–9], detecting cancer in mammograms [10, 11], analysing computer tomography scans [12–15], identifying brain tumours on magnetic resonance images [16], and predicting development of Alzheimer’s disease from positron emission tomography [17]. Applications have also been shown in pathology [18], identifying cancerous skin lesions [19–22], interpreting retinal imaging [23, 24], detecting arrhythmias [25, 26], and even identifying hyperkalaemia from electrocardiograms [27]. Furthermore, AI has aided in polyp detection from colonoscopy [28], improving genomics interpretation [29], identifying genetic conditions from facial appearance [30], and assessing embryo quality to maximise the success of in vitro fertilisation [31].

Analysis of the immense volume of data collected from electronic health records (EHRs) offers promise in extracting clinically relevant information and making diagnostic evaluations [32] as well as in providing real-time risk scores for transfer to intensive care [33], predicting in-hospital mortality, readmission risk, prolonged length of stay and discharge diagnoses [34], predicting future deterioration, including acute kidney injury [35], improving decision-making strategies, including weaning of mechanical ventilation [36] and management of sepsis [37], and learning treatment policies from observational data [38]. Proof-of-concept studies have aimed to improve the clinical workflow, including automatic extraction of semantic information from transcripts [39], recognising speech in doctor–patient conversations [40], predicting risk of failure to attend hospital appointments [41], and even summarising doctor–patient consultations [42].

Given this impressive array of studies, it is perhaps surprising that real world deployments of machine learning algorithms in clinical practice are rare. Despite this, we believe that AI will have a positive impact on many aspects of medicine. AI systems have the potential to reduce unwarranted variation in clinical practice, improve efficiency and prevent avoidable medical errors that will affect almost every patient during their lifetime [43]. By providing novel tools to support patients and augment healthcare staff, AI could enable better care delivered closer to the patient in the community. AI tools could assist patients in playing a greater role in managing their own health, primary care physicians by allowing them to confidently manage a greater range of complex disease, and specialists by offering superhuman diagnostic performance and disease management. Finally, through the detection of novel signals of disease that clinicians are unable to perceive, AI can extract novel insights from existing data. Examples include the identification of novel predictive features for breast cancer prognosis using stromal cells (rather than the cancer cells themselves) [44], predicting cardiovascular risk factors and sex from a fundus photograph [45], inferring blood flow in coronary arteries from cardiac computed tomography [46], detecting individuals with atrial fibrillation from ECG acquired during normal sinus rhythm [26], and using retinal imaging to assist an earlier diagnosis of dementia [47].

## The challenge of translation to clinical practice

### Retrospective versus prospective studies

While existing studies have encompassed very large numbers of patients with extensive benchmarking against expert performance, the vast majority of studies have been retrospective, meaning that they use historically labelled data to train and test algorithms. Only through prospective studies will we begin to understand the true utility of AI systems, as performance is likely to be worse when encountering real-world data that differ from that encountered in algorithm training. The limited number of prospective studies to date include diabetic retinopathy grading [48–50], detection of breast cancer metastases in sentinel lymph node biopsies [51, 52], wrist fracture detection [53], colonic polyp detection [28, 54], and detection of congenital cataracts [55]. Consumer technology is enabling enormous prospective studies, in relation to historical standards, through the use of wearables; for example, there is an ongoing study to detect atrial fibrillation in 419,093 consenting Apple watch owners [56].

### Peer-reviewed randomised controlled trials as an evidence gold standard

As is common in the machine learning community, many studies have been published on preprint servers only and are not submitted to peer-reviewed journals. Peer-reviewed evidence will be important for the trust and adoption of AI within the wider medical community. There are very few randomised controlled trials (RCTs) of AI systems to date; these include an algorithm to detect childhood cataracts with promising performance in a small prospective study [55] but less accurate performance compared to senior clinicians in a diagnostic RCT [57]; a single-blind RCT that showed a significantly reduced blind-spot rate in esophagogastroduodenoscopy [58]; an open, non-blinded randomised trial of an automatic polyp detection algorithm for diagnostic colonoscopy demonstrating a significant increase in detection of diminutive adenomas and hyperplastic polyps [59]; a simulated prospective, double-blind RCT of an algorithm to detect acute neurologic events [60]; and an unmasked RCT of a system to provide automated interpretation of cardiotocographs in labour that found no improvement in clinical outcomes for mothers or babies [61]. The final study is a cautionary example of how higher accuracy enabled by AI systems does not necessarily result in better patient outcomes [61]. Future studies should aim to use clinical outcomes as trial endpoints to demonstrate longer-term benefit, while recognising that algorithms are likely to result in changes of the sociocultural context or care pathways; this may necessitate more sophisticated approaches to evaluation [62].

High quality reporting of machine learning studies is critical. Only with full and clear reporting of information on all aspects of a diagnosis or prognosis model can risk of bias and potential usefulness of prediction models be adequately assessed. Machine learning studies should aim to follow best practice recommendations, such as the Transparent Reporting of a multivariable prediction model for Individual Prognosis Or Diagnosis (TRIPOD), designed to assist the reporting of studies that develop, validate or update a prediction model for either diagnostic or prognostic purposes [63]. In addition, a new version of the TRIPOD statement that is specific to machine learning prediction algorithms (TRIPOD-ML) is in development and will focus on the introduction of machine learning prediction algorithms, establishing methodological and reporting standards for machine learning studies in healthcare [64].

### Metrics often do not reflect clinical applicability

The term ‘AI chasm’ has been coined to reflect the fact that accuracy does not necessarily represent clinical efficacy [65]. Despite its universal use in machine learning studies, area under the curve of a receiver operating characteristic curve is not necessarily the best metric to represent clinical applicability [66] and is not easily understandable by many clinicians. As well as reporting sensitivity and specificity at a selected model operating point (required to turn the continuous model output into discrete decision categories), papers should include information about positive and negative predictive values. As no single measure captures all the desirable properties of a model, several measures are typically reported to summarise its performance. However, none of these measures ultimately reflect what is most important to patients, namely whether the use of the model results in a beneficial change in patient care [67].

Clinicians need to be able to understand how the proposed algorithms could improve patient care within a relatable workflow, yet most papers do not attempt to present such information; potential approaches to this have been suggested, including decision curve analysis, which aims to quantify the net benefit of using a model to guide subsequent actions [68]. To improve understanding, medical students and practising clinicians should be provided with an easily accessible AI curriculum to enable them to critically appraise, adopt and use AI tools safely in their practice.

### Difficulty comparing different algorithms

The comparison of algorithms across studies in an objective manner is challenging due to each study’s performance being reported using variable methodologies on different populations with different sample distributions and characteristics. To make fair comparisons, algorithms need to be subjected to comparison on the same independent test set that is representative of the target population, using the same performance metrics. Without this, clinicians will have difficulty in determining which algorithm is likely to perform best for their patients.

The curation of independent local test sets by each healthcare provider could be used to fairly compare the performance of the various available algorithms in a representative sample of their population. Such independent test sets should be constructed using an unenriched representative sample along with data that are explicitly not available to train algorithms. A supplementary local training dataset could be provided to allow fine tuning of algorithms prior to formal testing.

For researchers, comparison will become easier with the increasing availability of large, open datasets, allowing studies to benchmark their performance in a consistent manner.

### Challenges related to machine learning science

AI algorithms have the potential to suffer from a host of shortcomings, including inapplicability outside of the training domain, bias and brittleness (tendency to be easily fooled) [69]. Important factors for consideration include dataset shift, accidentally fitting confounders rather than true signal, propagating unintentional biases in clinical practice, providing algorithms with interpretability, developing reliable measures of model confidence, and the challenge of generalisation to different populations.

### Dataset shift

Particularly important for EHR algorithms, it is easy to ignore the fact that all input data are generated within a non-stationary environment with shifting patient populations, where clinical and operational practices evolve over time [70]. The introduction of a new predictive algorithm may cause changes in practice, resulting in a new distribution compared to that used to train the algorithm. Therefore, methods to identify drift and update models in response to deteriorating performance are critical. Mitigations to manage this effect include careful quantification of performance over time to proactively identify problems, alongside the likely requirement for periodical retraining. Data-driven testing procedures have been suggested to recommend the most appropriate updating method, from simple recalibration to full model retraining, in order to maintain performance over time [71].

### Accidentally fitting confounders versus true signal

Machine learning algorithms will use whatever signals are available to achieve the best possible performance in the dataset used. This may include the exploitation of unknown confounders that may not be reliable, impairing the algorithm’s ability to generalise to new datasets. For instance, in one classic example, a machine learning model did not learn the intrinsic difference between dogs and wolves, but instead learned that wolves are usually pictured standing on snow, while dogs usually appear on grass [72]. There are similar concerns in healthcare. In one study, an algorithm was more likely to classify a skin lesion as malignant if an image had a ruler in it because the presence of a ruler correlated with an increased likelihood of a cancerous lesion [19]. The presence of surgical skin markings have also been shown to falsely increase a deep learning model’s melanoma probability scores and hence false positive rate [73]. In another study, hip fracture detection was found to be aided by confounders, including the scanner model and scans marked ‘urgent’ [74]. Another algorithm for detection of pneumonia on chest x-rays was able to accurately identify hospital equipment and department, learning an association between a portable x-ray machine and pneumonia [75]. Ongoing work is required to understand the specific features being learned by neural networks and will be critical for generalisation across multiple healthcare settings.

### Challenges in generalisation to new populations and settings

The majority of AI systems are far from achieving reliable generalisability, let alone clinical applicability, for most types of medical data. A brittle model may have blind spots that can produce particularly bad decisions. Generalisation can be hard due to technical differences between sites (including differences in equipment, coding definitions, EHR systems, and laboratory equipment and assays) as well as variations in local clinical and administrative practices.

To overcome these issues, it is likely that a degree of site-specific training will be required to adapt an existing system for a new population, particularly for complex tasks like EHR predictions. Methods to detect out-of-distribution inputs and provide a reliable measure of model confidence will be important to prevent clinical decisions being made on inaccurate model outputs. For simpler tasks, including medical image classification, this problem may be less crucial and overcome by the curation of large, heterogenous, multi-centre datasets [14]. Generalisation of model operating points may also prove challenging across new populations, as illustrated in a recent study to detect abnormal chest radiographs, where specificity at a fixed operating point varied widely, from 0.566 to 1.000, across five independent datasets [5].

Proper assessment of real-world clinical performance and generalisation requires appropriately designed external validation involving testing of an AI system using adequately sized datasets collected from institutions other than those that provided the data for model training. This will ensure that all relevant variations in patient demographics and disease states of target patients in real-world clinical settings are adequately represented in the system where it will be applied [76]. This practice is currently rare in the literature and is of critical concern. A recent systematic review of studies that evaluated AI algorithms for the diagnostic analysis of medical imaging found that only 6% of 516 eligible published studies performed external validation [77].

### Algorithmic bias

Intertwined with the issue of generalisability is that of discriminatory bias. Blind spots in machine learning can reflect the worst societal biases, with a risk of unintended or unknown accuracies in minority subgroups, and there is fear over the potential for amplifying biases present in the historical data [78]. Studies indicate that, in some current contexts, the downsides of AI systems disproportionately affect groups that are already disadvantaged by factors such as race, gender and socioeconomic background [79]. In medicine, examples include hospital mortality prediction algorithms with varying accuracy by ethnicity [80] and algorithms that can classify images of benign and malignant moles with accuracy similar to that of board-certified dermatologists [19, 81], but with underperformance on images of lesions in skin of colour due to training on open datasets of predominantly fair skinned patients. The latter is particularly concerning as patients with skin of colour already present with more advanced dermatological diseases and have lower survival rates than those with fair skin [82].

Algorithmic unfairness can be distilled into three components, namely (1) model bias (i.e. models selected to best represent the majority and not necessarily underrepresented groups), (2) model variance (due to inadequate data from minorities), and (3) outcome noise (the effect of a set of unobserved variables that potentially interacts with model predictions, avoidable by identifying subpopulations to measure additional variables) [80]. A greater awareness of these issues and empowering clinicians to participate critically in system design and development will help guide researchers to ensure that the correct steps are taken to quantify bias before deploying models. Algorithms should be designed with the global community in mind, and clinical validation should be performed using a representative population of the intended deployment population. Careful performance analysis by population subgroups should be performed, including age, ethnicity, sex, sociodemographic stratum and location. Analysis to understand the impact of a new algorithm is particularly important, i.e. if the spectrum of disease detected using the AI system differs from current clinical practice, then the benefits and harms of detecting this different spectrum of disease must be evaluated. In mammography, this might be the detection of less severe ductal carcinoma in situ, potentially resulting in increased treatment with little benefit in outcomes. Prospective pilots within healthcare systems should be undertaken to understand the product characteristics and identify potential pitfalls in practical deployment.

### Susceptibility to adversarial attack or manipulation

Algorithms have been shown to be susceptible to risk of adversarial attack. Although somewhat theoretical at present, an adversarial attack describes an otherwise-effective model that is susceptible to manipulation by inputs explicitly designed to fool them. For example, in one study, images of benign moles were misdiagnosed as malignant by adding adversarial noise or even just rotation [83].

### Logistical difficulties in implementing AI systems

Many of the current challenges in translating AI algorithms to clinical practice are related to the fact that most healthcare data are not readily available for machine learning. Data are often siloed in a multitude of medical imaging archival systems, pathology systems, EHRs, electronic prescribing tools and insurance databases, which are very difficult to bring together. Adoption of unified data formats, such as Fast Healthcare Interoperability Resources [84], offer the potential for better aggregation of data, although improved interoperability does not necessarily fix the problem of inconsistent semantic coding in EHR data [85].

### Achieving robust regulation and rigorous quality control

A fundamental component to achieving safe and effective deployment of AI algorithms is the development of the necessary regulatory frameworks. This poses a unique challenge given the current pace of innovation, significant risks involved and the potentially fluid nature of machine learning models. Proactive regulation will give confidence to clinicians and healthcare systems. Recent U.S. Food and Drug Administration guidance has begun developing a modern regulatory framework to make sure that safe and effective artificial intelligence devices can efficiently progress to patients [86].

It is also important to consider the regulatory impact of improvements and upgrades that providers of AI products are likely to develop throughout the life of the product. Some AI systems will be designed to improve over time, representing a challenge to traditional evaluation processes. Where AI learning is continuous, periodic system-wide updates following a full evaluation of clinical significance would be preferred, compared to continuous updates which may result in drift. The development of ongoing performance monitoring guidelines to continually calibrate models using human feedback will support the identification of performance deficits over time.

### Human barriers to AI adoption in healthcare

Even with a highly effective algorithm that overcomes all of the above challenges, human barriers to adoption are substantial. In order to ensure that this technology can reach and benefit patients, it will be important to maintain a focus on clinical applicability and patient outcomes, advance methods for algorithmic interpretability, and achieve a better understanding of human–computer interactions.

### Algorithmic interpretability is at an early stage but rapidly advancing

While AI approaches in medicine have yielded some impressive practical successes to date, their effectiveness is limited by their inability to ‘explain’ their decision-making in an understandable way [87]. Even if we understand the underlying mathematical principles of such models, it is difficult and often impossible to interrogate the inner workings of models to understand how and why it made a certain decision. This is potentially problematic for medical applications, where there is particular demand for approaches that are not only well-performing, but also trustworthy, transparent, interpretable and explainable [88].

Healthcare offers one of the strongest arguments in favour of explainability [88, 89]. Given the combination of the devastating consequences of unacceptable results, the high risk of unquantified bias that is difficult to identify a priori, and the recognised potential for models to use inappropriate confounding variables, explainability enables system verification. This improves experts’ ability to recognise system errors, detect results based upon inappropriate reasoning, and identify the work required to remove bias. In addition, AI systems are trained using large numbers of examples and may detect patterns in data that are not accessible to humans. Interpretable systems may allow humans to extract this distilled knowledge in order to acquire new scientific insights. Finally, recent European Union General Data Protection Regulation legislation mandates a ‘right to explanation’ for algorithmically generated user-level predictions that have the potential to ‘significantly affect’ users; this suggests that there must be a possibility to make results re-traceable on demand [88].

At present, a trade-off exists between performance and explainability. The best performing models (e.g. deep learning) are often the least explainable, whereas models with poorer performance (e.g. linear regression, decision trees) are the most explainable. A key current limitation of deep learning models is that they have no explicit declarative knowledge representation, leading to considerable difficulty in generating the required explanation structures [90]. Machine learning methods that build upon a long history of research in traditional symbolic AI techniques to allow for encoding of semantics of data and the use of ontologies to guide the learning process may permit human experts to understand and retrace decision processes more effectively [91, 92]. One recent approach replaced end-to-end classification with a two-stage architecture comprising segmentation and classification, allowing the clinician to interrogate the segmentation map to understand the basis of the subsequent classification [24].

If ‘black box’ algorithms are to be used in healthcare, they need to be used with knowledge, judgement and responsibility. In the meantime, research into explainable AI and evaluation of interpretability is occurring at a rapid pace [93]. Explainable AI approaches are likely to facilitate faster adoption of AI systems into the clinical healthcare setting, and will help foster vital transparency and trust with their users.

### Developing a better understanding of interaction between human and algorithm

We have a limited but growing understanding of how humans are affected by algorithms in clinical practice. Following the U. S. Food and Drug Administration approval of computer-aided diagnosis for mammography in the late 1990s, computer-aided diagnosis was found to significantly increase recall rate without improving outcomes [94]. Excessive warnings and alerts are known to result in alert fatigue [94, 95]. It has also been shown that humans assisted by AI performed better than either alone in a study of diabetic retinopathy screening [96, 97]. Techniques to more meaningfully represent medical knowledge, provide explanation and facilitate improved interaction with clinicians will only improve this performance further. We need to continue gaining a better understanding of the complex and evolving relationship between clinicians and human-centred AI tools in the live clinical environment [98].

## Conclusion

Recent advances in artificial intelligence present an exciting opportunity to improve healthcare. However, the translation of research techniques to effective clinical deployment presents a new frontier for clinical and machine learning research. Robust, prospective clinical evaluation will be essential to ensure that AI systems are safe and effective, using clinically applicable performance metrics that go beyond measures of technical accuracy to include how AI affects the quality of care, the variability of healthcare professionals, the efficiency and productivity of clinical practice and, most importantly, patient outcomes. Independent datasets that are representative of future target populations should be curated to enable the comparison of different algorithms, while carefully evaluating for signs of potential bias and fitting to unintended confounders. Developers of AI tools must be cognisant of the potential unintended consequences of their algorithms and ensure that algorithms are designed with the global community in mind. Further work to improve the interpretability of algorithms and to understand human–algorithm interactions will be essential to their future adoption and safety supported by the development of thoughtful regulatory frameworks.

## Data Availability

Not applicable.

## Abbreviations

AI: artificial intelligence
EHRs: electronic health records
RCT: randomised controlled trial
TRIPOD: Transparent Reporting of a multivariable prediction model for Individual Prognosis Or Diagnosis
